# Magnetophoretic transport of functionalised iron-oxide nanoparticles through biomimetic hydrogels and extracellular matrix

**DOI:** 10.1039/d6na00088f

**Published:** 2026-05-12

**Authors:** Stephen Lyons, Katie McGarry, Aline F. Miller, Rinki Singh, Aoife Morrin, Dermot F. Brougham

**Affiliations:** a School of Chemical Sciences, Insight Research Ireland Centre for Data Analytics, Dublin City University Dublin 9 Ireland; b School of Chemistry, University College Dublin Belfield Dublin 4 Ireland dermot.brougham@ucd.ie; c Department of Materials & Manchester Institute of Biotechnology (MIB), School of Natural Sciences, Faculty of Science and Engineering, The University of Manchester UK

## Abstract

Magnetic nanoparticles show promise for applications including targeted drug delivery, contrast-enhanced imaging, and theranostics, but their efficacy is hindered by limited understanding of nanoparticle–tissue interactions that influence transport through biological tissue under magnetic field gradients. Here, we assess the particle size, surface chemistry, and magnetic field gradient dependence of magnetophoretic transport of magnetic nanoparticles (MNPs) through tissue models of increasing complexity/biological relevance. In all cases linear particle transits were observed through the gels, with progressively increasing velocity for higher gradients. The effect of particle size on velocity is negligible at low and intermediate gradient, but at higher gradient the larger MNPs have higher velocity (*p*-value 0.002). Scaled velocities, *v*_exp_ × *d*_hyd_, were found to correct for hydrodynamic size-induced differences in drag, enabling identification of MNP–matrix interactions that arise from the particle surface functionalisation used; arginine- (Arg-; positive), citrate- (Cit-; negative) and polyethylene glycol (PEG-; neutral). In agarose *v*_exp_ × *d*_hyd_ is higher for Arg- and lower for Cit-, as compared to PEG-MNPs, due to increased and reduced flux, respectively, at negatively charged pore restrictions. This effect is eliminated at very high gradient (pore deformation), or on increasing the ionic strength (reduced electrostatic interactions). In contrast in agarose–collagen hydrogels for Cit-MNPs and particularly Arg-MNPs net attraction to the matrix, due to residual electrostatic interactions with the collagenous component, is evident even at high ionic strength. In ECM relatively slow scaled velocity is observed, despite the open pore structure. Cit-MNPs are particularly hindered, suggesting the residual electrostatic interactions are stronger in this case. The findings contribute to understanding of matrix–particle interactions in models of biological tissue, informing material design for effective MNP transport in targeted nanomaterial-based diagnostics and treatment applications.

## Abstract 2

Magnetic nanoparticles have potential in targeted drug delivery and contrast-enhanced imaging, but their application is hindered by limited understanding of nanoparticle–tissue interactions under magnetic field gradients that determine transport.

## Graphical abstract

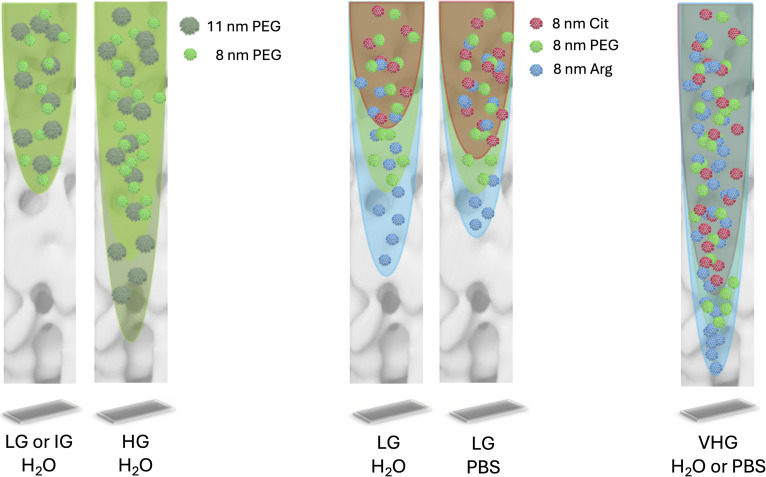

## Introduction

Transport of magnetic iron oxide nanoparticles (MNPs) through tissue under the influence of magnetic field gradients is key for development of magnetic drug delivery systems,^1–3^ target capture methods^4^ and photodynamic^3^ and hyperthermic cancer treatments.^5,6^ Similarly, MNP transport (and subsequent capture) through artificial bio-inspired matrices is a key pre-concentration step in established RNA amplification/detection protocols,^7^ and is relevant for development of biomarker quantification.^8,9^ Hydrogels have also been studied as models for nanoparticle transport in tissue.^9–13^ Dynamic magnetic cell culture systems are emerging with on-chip formats that can provide magnetic stimulus in perfused environments. For instance, a recent study exploited a tumour-on-a-chip format to evaluate brain tissue penetration and compression by MNPs in a field gradient.^14^ However, the impact of matrix–MNP interactions in biological and biomimetic environments during magnetophoretic transport is not fully understood and remains key to exploitation of these technologies.

The relevant literature comprises a report from Holligan *et al.*^12^ of water dispersed 10 nm (diameter) MNPs placed onto agarose gels which accelerate in the presence of a field gradient. Agarose hydrogels are often used as mimics to model behaviour of *in vivo* tissues. As the drag force rapidly increases to match the magnetic force linear transport was observed, with constant terminal velocity in the range of 0.2–0.5 mm h^−1^, reported when transport was driven by a neodymium alloy magnet (providing gradients of 30–50 T m^−1^). The slow transit was ascribed to the tortuosity of the MNP paths through the porous matrix. Subsequently, linear transport, with similar velocities under similar conditions, was reported by Kuhn *et al.*, for larger (279 and 800 nm) nanocomposite particles in transit through purified extracellular matrix.^15^ The particles were composed of silica/dextran with superparamagnetic MNPs embedded at 75 wt%. Magnetic transport of similar particles (>100 nm, with embedded superparamagnetic MNPs) through excised tissue was also assessed by Kulkarni *et al.*^16^ In this phenomenological study penetration depths were measured at fixed times the velocities were, again, in the 0.2–0.7 mm h^−1^ range.

More recently we evaluated the transport through agarose hydrogels of fully dispersed spherical MNPs formed using the same magnetic cores, but with different surface chemistries; neutral (polyethylene glycol, PEG-); anionic (citrate, Cit-), and; cationic (3-aminopropyl)triethoxysilane, APTES-stabilised.^13^ Linear transits were again observed in all cases, and the velocity was found to be independent of MNP concentration (up to 2 mg mL^−1^), while the particles in the recovered suspensions remained fully dispersed with unchanged *d*_hyd_, and so were unmodified by the transit. The magnetophoretic velocity was found to be determined only by matrix tortuosity (wt% agarose) and particle hydrodynamic size, *d*_hyd_, once electrostatic MNP–matrix interactions were suppressed. That was the case for PEGylated MNPs (for which there are minimal electrical double layers, EDLs) and for electrostatically-stabilised MNPs when high ionic strength (IS) media was used (the EDLs collapse). In the absence of electrostatic interactions and treating the medium as a continuum, the balance of magnetic and drag forces^12,15^ gives the following relation (rearranged here for illustration):1
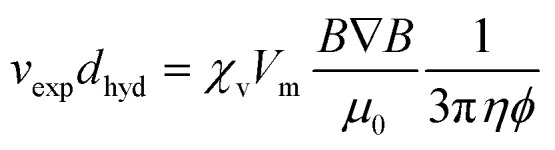
where *v*_exp_ is the MNP velocity, *d*_hyd_ the hydrodynamic diameter (taken from dynamic light scattering, DLS); *χ*_V_ the volumetric magnetic susceptibility; *V*_m_ the volume of superparamagnetic material in a single particle (from transmission electron microscopy, TEM); *B* the magnetic field strength, ∇*B* the gradient of the magnetic field; *µ*_0_ the permeability of free space; *ϕ* is a scaling factor characterising the tortuosity of the pathway for MNP transport (1/*ϕ* → 0 for increasingly tortuous paths, and 1/*ϕ* → 1 for free diffusion), and; *η* is the viscosity along that path. For a given matrix composition all the terms on the right should be constant. We found that for any wt% agarose (*i.e.* for a given tortuosity) the measured values of *v*_exp_ × *d*_hyd_ for different sized MNPs were indeed the same, once the EDLs were collapsed. This product, the ‘scaled velocity’, effectively corrects for differences in drag, enabling comparisons of the transport of different sized particles. *v*_exp_ × *d*_hyd_ is thus more instructive than the unscaled magnetophoretic velocity.

We observed that when electrostatic interactions were present *v*_exp_ × *d*_hyd_ was modulated;^12,13^ positively charged MNPs travelled faster, and negatively charged MNPs slower, than predicted. Our interpretation was that negatively charged MNPs are forced to the centre of the negatively charged pore restrictions (openings) in the agarose matrix and so progress more slowly, while positively charged MNPs are attracted by the chains at the restrictions so their flux is higher. These findings are akin to the exclusion enrichment (EE) effect known for transport of charged molecules through hard nano-pores.^17,18^

In this work we examine magnetophoretic transport of MNPs through tissue models of progressively increasing complexity/biological relevance; (i) agarose hydrogels at low and high IS; (ii) agarose–collagen hydrogels expanded in synthetic interstitial fluid (ISF_syn_), and; (iii) cultured cell-derived decellularized extracellular matrix (ECM). Next we briefly describe these selected hydrogels.

It is known that gelation in agarose, a galactopyranose-based polysaccharide, occurs on cooling when random coils form single or double helices which themselves aggregate into ordered structures of bundled double helices interconnected with flexible chains.^19^ The matrix is usually isotropic, with porosity in the 100–300 nm range.^20^ It is a cheap, non-toxic, material with sensitively tunable stiffness making it ideal for many cell culture scaffold applications that do not require more bioactive/biomimetic environment.

At the other extreme of complexity is the extracellular matrix (ECM), which provides the cellular microenvironment. It is composed of a fibrous network, of collagen and elastin, and glycoproteins including fibronectin, glycosaminoglycan (typically hyaluronan) decorated protein-proteoglycans, and laminin forming a supra-structure whose architecture is dictated by the component molecules and the cells that are present.^21^ The extracellular matrix provides mechanical/structural support and is the source of signalling molecules that determine cell proliferation, differentiation and migration. It can be extracted, decellularised and purified and is commercially available. The diffusion of non-magnetic nanoparticles through ECM has been studied experimentally,^22^ modelled^23^ and reviewed.^21^ Despite the presence of nanoscale pores, diffusion of positively or negatively charged particles is effectively suppressed^24^ while uncharged, typically PEGylated, particles^22^ easily pass through the matrix. Attractive interactions between charged particles and chains, primarily the heparan sulphate, were identified as an important factor in this barrier.

An interesting alternative hydrogel can be formed by incorporating agarose into networks of collagen I, in which the polysaccharide forms a dense mesh between the collagen fibres. Increasing agarose content has been shown to dramatically increase matrix elasticity, by structurally coupling and reinforcing the fibers but with minimal fibre re-organisation.^25^ Hence agarose–collagen hydrogels provide stiffness that is tunable through the composition. These composites are attractive for 3D cell culture and tissue engineering applications, providing both insights into deformation under indentation and useful models for studying cell infiltration.^25–27^

Here we evaluate the particle- and field gradient-dependent factors that determine MNP transport for a range suspensions (of particles with different sizes and with cationic, anionic and neutral surface chemistries) through the selected hydrogels under conventional, and higher magnetic field gradients than previously reported. The goals were to evaluate, and where possible separate, the impact of electrostatics from other contributions, and to add to the growing understanding of matrix–particle interactions in biomimetic environments which is key to development of diagnostics and treatments.

## Experimental

### Reagents and equipment

Iron acetylacetonate (14024-18-1), benzyl alcohol (100-51-6), (3-glycidyloxypropyl)trimethoxysilane (GLYMO) (2530-83-8), end terminal aminated polyethylene glycol (PEG) MW1000 and MW2000, (25322-68-3), acetone (67-64-1), sodium citrate tribasic (iii) (6132-04-3), chloroform (67-66-3), tetrahydrofuran (THF) (109-99-9), potassium hydroxide (1310-58-3), hydrochloric acid (7647-01-0), agarose (9012-36-6) (low electroendosmosis (EEO), unless otherwise specified) (0.09–0.13), aqueous ammonia (1336-21-6), 4-(2-hydroxyethyl)piperazine-1-ethanesulfonic acid (HEPES) (7365-45-9) and PBS tablets (78392) were all purchased from Sigma. l-Arginine (74-79-3) was purchased from Biochemika. Collagen Type IV from bovine skin (9007-34-5), ECM gel liquid prepared from Engelbreth–Holm–Swarm murine sarcoma (E1270), isopropyl alcohol (67-63-0) were purchased from Sigma.

The low field gradient (LG) was generated by placing the samples on a flat corner of a 50 × 25 mm N52 neodymium magnet (F335-N52). This provides a gradient of *c.* 45 T m^−1^, and a field at the surface of 0.55 T. This is a conventional, mature technology and the gradients achieved are very well characterised. For very high field gradient (VHG) a custom-built magnet (GIAMAG Technologies) was used. This magnet is comprised of several strips of neodymium (providing a strong magnetic field of *c.* 1.1 T) encased in a shielding material. This concentrated gradient of *c.* 236 T m^−1^ is focused through a circular (20 mm diameter) opening at the top of the magnet. There is some homogeneity and variability in the stray field, but the gradient generated is ∼5 times that of the N52. The gradients achieved with this design approach were confirmed by GIAMAG using measurement and finite element modelling. The intermediate gradient (IG) was achieved using the GIAMAG and reducing the gradient at the sample by lifting it using a flat parafilm spacer, fashioned to fill the 20 × 6 mm cylindrical opening. The high gradient (HG) was achieved using the GIAMAG with a reduced static field of *c.* 0.30 T (with no spacer). The estimated (given the noted uncertainties) magnetic forces (*B*·∇*B* values) for the study were 260 ± 25, 155 ± 30, 82 ± 16 and 25 ± 5 T^2^ m^−1^ for VHG, HG, IG and LG, respectively. These values are guidelines only as they reflect measurement in air rather than the forces experienced by MNPs in gels, when held in a vial at the pole face.

### Materials synthesis and methodology

#### MNP synthesis

Spherical MNPs in the core size range of 8–9 nm were synthesised using an adaptation of the Pinna method,^28^ described by us previously.^29^ Briefly, the MNPs were formed by mixing iron acetylacetonate (1 g) with benzyl alcohol (20.0 mL). This mixture was placed into a G30 glass test tube and microwave digested for 3 h at 200 °C under pressure (18 bar). The resulting suspension was 50.0 mg mL^−1^ of γ-Fe_2_O_3_ MNPs.

To prepare 8 nm MNPs, an adapted^29^ version of the surfactant free thermal decomposition method^28^ was used. Fe(acac)_3_ (2.00 g) was added to a 100 mL three neck round bottom flask (RBF), benzyl alcohol (BA, 40 mL) was then added to the RBF and the mixture was stoppered and gently vortexed for 1–2 min. The RBF was placed in a heating mantle and attached to a solvent trap and the reaction mixture was then degassed for *c.* 20 min with N_2_. The reaction mixture was then heated to reflux, *c.* 210 °C, for 7 h and left to cool to RT. The black NP mixture was washed three times with BA (50 mL), until the BA following magnetic decantation was clear. The final slurry was suspended in BA (10 mL), and flushed gently with N_2_, before storage at 4 °C.

To prepare 11 nm MNPs, following the final resuspension the liquid was transferred to a clean flame dried RBF and the BA decanted. Fe(acac)_3_ (2.00 g) was then added, and BA (40 mL) and the reaction was refluxed at 210 °C for 7 h as before. The workup was as for 8 nm MNPs.

The core diameters for each batch were determined by transmission electron microscopy (TEM) carried out using a FEI Tecnai G2 20 TWIN 200 kV. In each case, 5 µL of suspension was pipetted onto a carbon TEM grid and allowed to air dry overnight before imaging.

The magnetic properties of the materials prepared with this approach have previously been reported by ourselves. They have also been shown to be reproducible batch-to-batch and to be stable in suspension over several weeks,^29^*i.e.* for far longer than the interval prior to the magnetophoretic measurements in this study. The MNPs are superparamagnetic at RT, as shown by the absence of coercivity using DC-magnetometry.^13^ We have also shown, using X-ray absorption spectroscopy, that the phase formed most closely resembles maghemite, γ-Fe_2_O_3_.^30^

#### MNP functionalisation

To functionalise the MNP surface with PEG or arginine, GLYMO-functionalised MNPs were first synthesised according to ref. 29. Briefly, 2.0 mL of MNPs (1.0 mg mL^−1^) was added to a vial with 4 mL of acetone. This caused the MNPs to precipitate out of the benzyl alcohol. The glass vial was then placed on a magnet to retain the MNPs while the benzyl alcohol/acetone mixture was removed. This step was repeated 3 times to ensure all benzyl alcohol was removed. GLYMO (50.0 µL) was dissolved with chloroform (2 mL) and added to the MNP material. This mixture was then placed on a plate shaker at 400 rpm for 24 h. THF (2 mL) was used to remove excess GLYMO by magnetic separation after agitation.

To prepare PEG-MNPs,^29^ PEG (7.0 µL) was dissolved in THF (2 mL) and this was added to the GLYMO-MNP material. KOH (50 µL, 1.0 M) was then added to precipitate out the MNPs. The resulting PEG-MNPs were then dispersed in deionised (DI) H_2_O at desired concentration. The starting molecular weight of the PEG used was either 1000 or 2000. GLYMO-MNPs were also functionalised with arginine in the same manner, whereby arginine (0.005 g) was dissolved in DI H_2_O (2 mL) and added to the GLYMO-MNPs in place of PEG.

Citrate-MNPs were synthesised starting with the precipitate from benzyl alcohol, as before. Once all benzyl alcohol was removed from the bare MNPs, sodium citrate in DI H_2_O (0.6 g L^−1^) was added to the MNP material to yield a ∼1.0 mg mL^−1^ MNP suspension. This suspension was then placed on a plate shaker at 400 rpm for approx. 4 h. The functionalisation was deemed complete when the suspension turned from blue–black to a dark brown colour.

### Gel expansion solution preparation

Three solutions were used to expand agarose and agarose–collagen; DI H_2_O, 0.1 M PBS (IS 0.14 M) and synthetic interstitial fluid (ISF_syn_). ISF_syn_ was composed of CaCl_2_ (2 mM), HEPES (10 mM), glucose (5.5 mM), KCl (3.5 mM), magnesium sulphate (0.7 mM), NaCl (123 mM), monosodium phosphate (1.49 mM), and saccharose (0.9 mM); pH 7.4, IS 0.16 M. Both ionic expansion solutions, PBS and ISF_syn_ are considered to have approx. equivalent IS and consideration is limited to IS composition only for the purposes of this work.

### Agarose gel preparation

To prepare agarose gel (0.3% w/v), 0.06 g of agarose was weighed out and added to DI H_2_O, PBS (IS 0.14 M) or ISF_syn_ (IS 0.16 M), as specified, to give gels referred to as agarose (H_2_O), agarose (PBS), agarose (ISF_syn_). This mixture was heated and stirred until the agarose had fully dissolved. Once the agarose solution was transparent, glass vials (53 × 16 × 16 mm) were filled with agarose solution to a depth of 6 mm and were left to cool at room temperature for 1 h. The vials were then capped and left to solidify overnight at 4 °C.

### Agarose–collagen gel preparation

Agarose–collagen (H_2_O) or agarose–collagen (ISF_syn_) were prepared by first dissolving agarose in DI H_2_O or ISF_syn_, respectively as described above, and 600 µL of solution were added to a glass vial (instead of 700 µL). Solutions were kept in the oven for 2 h at 37 °C and 100 µL of stock collagen solution (1 mg mL^−1^) was then added to a vial and vortexed for 30 s. The glass vial was then placed back into the oven at 37 °C for 1 h to induce self-assembly of the collagen fibres.^26^ Gels were removed from the oven and left to solidify for 1 h at room temperature. Vials were refrigerated at 4 °C until required. All gels were allowed to reach room temperature before use.

### ECM gel preparation

Prior to handling ECM, all workplace surfaces were washed down with isopropyl alcohol to ensure a sterile environment. ECM liquid was removed from the freezer and thawed overnight at 4 °C. 700 µL of the solution was transferred to a 7 mL glass vial. The vial was stored for 12 h at 4 °C to allow release of trapped air. The ECM was then incubated at 37 °C for 40 min to induce ECM polymerisation by self-assembly processes. ECM gels were used within 24 h of being prepared to avoid degradation. All glassware was treated with methanol to ensure sterility to avoid contamination.

### Measurement of MNP velocity

Measurement of MNP velocity (*v*_exp_) was carried out as previously described.^13^ Briefly, functionalised MNPs, as specified, at a concentration of ∼1.0 mg mL^−1^ were prepared. Gels (6 cm depth) were prepared in glass vials as described above, and placed onto a flat corner of the neodymium magnet, where the magnetic force was strongest, or onto/above the centre of the GIAMAG, Fig. S1. Then 100 µL of MNPs (1 mg mL^−1^) was pipetted onto the top surface of the gel to form an even layer of ∼1 mm thickness covering the entire upper gel surface (176.6 mm^2^), and the vials were capped. The MNPs were observed to migrate towards the base of the vial under the influence of the magnetic field over time Fig. S2. The vials were imaged every 30 min using a standard commercial digital camera. ImageJ software was used to process the images to track the motion of the MNP front in order to calculate the experimental MNP velocity (*v*_exp_).

### SEM

Selected images of agarose (H_2_O) and agarose–collagen (H_2_O) surface morphologies examined using SEM (Hitachi S-3400N) after freeze-drying samples at −58 °C for 48 h in a lyophiliser. Prior to lyophilisation, the gels were immersed in liquid nitrogen. Samples were mounted on carbon tape and gold-sputtered before analysis.

## Results & discussion

### Magnetophoretic transport of functionalised MNPs in agarose hydrogels

**Fig. 1:**
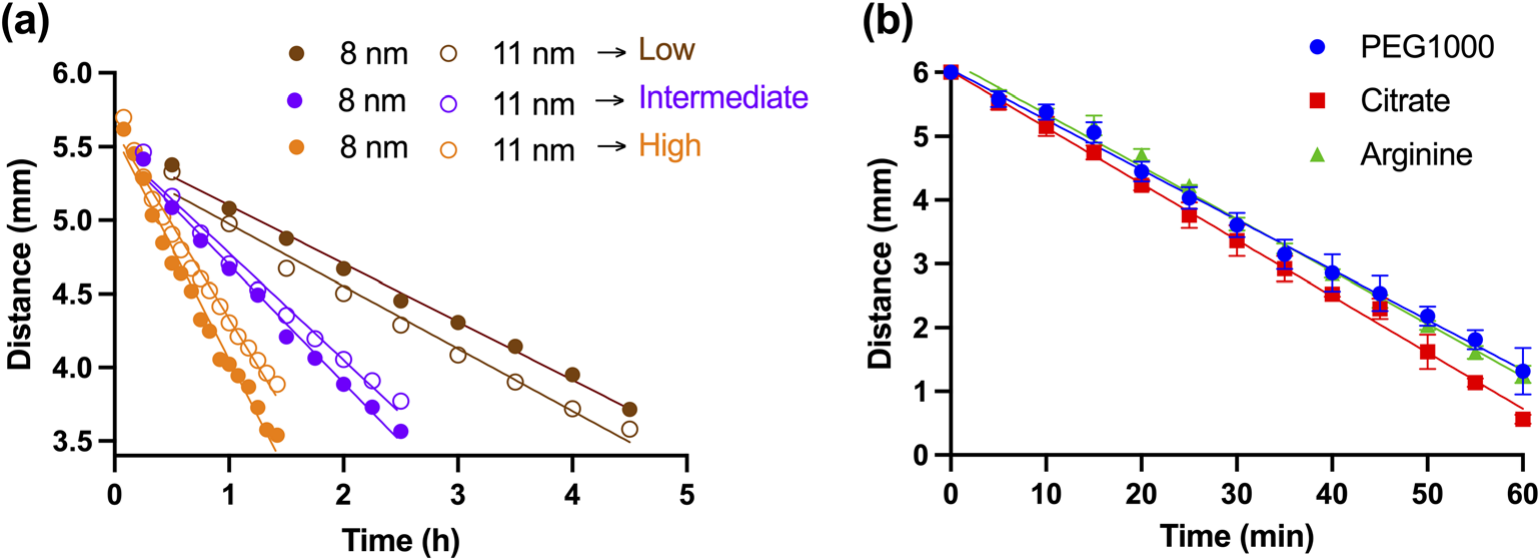
Representative magnetophoretic transport of magnetic nanoparticle suspensions (*n* = 3, at ∼1.0 mg mL^−1^ in DI H_2_O) through agarose (H_2_O), 0.3 % w/v. (a) PEG2000-MNPs; of size 8 nm (open data markers) and 11 nm (closed markers) using HG (
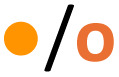
), IG (
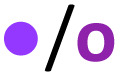
) and LG (
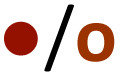
) magnetic field gradients; (b) 8 nm MNPs with different surface chemistry; PEG1000- (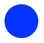); citrate- (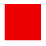); and arginine-MNPs (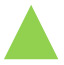), all using VHG.

Magnetophoretic transport was observed on pipetting the MNP suspensions onto the top of the (0.3% w/v) agarose hydrogels in the presence of a magnetic field gradient, *i.e.* with the gels (of 6 cm depth) positioned on top of a permanent magnet, see Methods. The subsequent transits of the MNP fronts were observed photographically and were found to be linear with time (constant velocity) for the four studied gradient strengths, for all particle sizes and surface chemistries, Fig. 1 and Tables 1 and 2. This demonstrates the generality of the linear behaviour. Given that the magnetic force follows an inverse square dependence with distance from the pole face, the retention of linearity during the entire transit may be explained by a viscoelastic matrix response.^9^ The forces opposing transport (including electrostatic interactions at pore restrictions, viscous drag within confined pores, elastic resistance of the network and local rearrangement of the gel structure path tortuosity) increase proportionally to the magnetic force applied, resulting in the observed constant velocities.

**Table 1:** Measured magnetophoretic velocities, *v*_exp_, and scaled velocities, *v*_exp_ × *d*_hyd_ for transport of 8 and 11 nm PEG2000-MNPs^a^ through agarose (H_2_O)

Size, *d* (nm)	Gradient	*v*_exp_ b (mm h^−1^)	Fractional change^c^	*v*_exp_ × *d*_hyd_ (×10^6^ mm^2^ h^−1^)
8	LG	0.42 (±0.02)	1.00	7.90
	IG	0.73 (±0.04)	+1.74	13.72
	HG	1.28 (±0.12)	+3.05	24.06
11	LG	0.40 (±0.01)	1.00	8.04
	IG	0.76 (±0.14)	+1.90	15.28
	HG	1.54 (±0.28)	+3.85	30.95

a 8 nm PEG2000-MNPs; *d*_TEM_ 8.1 ± 2.9 nm, *d*_hyd_ 18.8 nm, PDI 0.11. 11 nm PEG2000-MNPs; *d*_TEM_ 10.5 ± 2.9 nm, *d*_hyd_ 20.1 nm, PDI 0.20. Suspensions were at ∼1 mg mL^−1^, 0.3 % w/v.

b *v*_exp_ values determined by linear regression; *R*^2^ > 0.98 for all data sets; *n* = 3.

c Fractional change in *v*_exp_ (and also in *v*_exp_ × *d*_hyd_) relative to at low gradient.

**Table 2:** Scaled velocities (*v*_exp_ × *d*_hyd_) for low and very high magnetic field gradients for 8 nm PEG1000-, Arg-, and Cit-MNP suspensions through agarose (H_2_O) and agarose (PBS), as the isotonic condition. See Table S1 for *v*_exp_ values and measurement error

Surface chemistry	LG			VHG		
	Agarose (H_2_O)^b^	Agarose (PBS)		Agarose (H_2_O)	Agarose (PBS)	
	*v*_exp_·*d*_hyd_ (×10^6^ mm^2^ h^−1^)	*v*_exp_·*d*_hyd_ (×10^6^ mm^2^ h^−1^)	Fractional change^c^	*v*_exp_·*d*_hyd_ (×10^6^ mm^2^ h^−1^)^b^	*v*_exp_·*d*_hyd_ (×10^6^ mm^2^ h^−1^)	Fractional change^c^
PEG1000^a^	8.9	8.9	1.00	113	114	1.01
Arginine^a^	9.8	9.0	0.92	132	133	1.01
Citrate^a^	7.7	8.2	1.06	64.7	65.7	1.02

a PEG1000-MNPs from Batch 2, *d*_TEM_ 8.9 ± 0.8 nm (*d*_hyd_ 24.1 nm, PDI 0.16); Arg-MNPs (28.0 nm, 0.16); Cit-MNPs (12.1 nm, 0.17), for ∼1 mg mL^−1^.

b Values taken from ref. 13*n* = 4; *R*^2^ > 0.98 for all data sets.

c Fractional change in *v*_exp_·*d*_hyd_ relative to agarose (H_2_O).

Firstly, we assessed the impact of gradient strength and particle size, and so particle moment (effectively, *χ*_V_ × *V*_m_), on magnetophoretic transport through agarose expanded in DI H_2_O, or agarose (H_2_O). Two sizes of PEG2000 Da grafted MNPs, labelled 8 and 11 nm here, were evaluated. The suspension of smaller MNPs had *d*_TEM_ 8.1 ± 2.9 nm, and *d*_hyd_ 18.8 nm, PDI 0.11 (by DLS), and the larger had *d*_TEM_ 10.5 ± 2.9 nm, *d*_hyd_ 20.1 nm, PDI 0.20. The magnetophoresis data acquired for three gradient strengths is shown in Fig. 1a and Table 1.

**Scheme 1:**
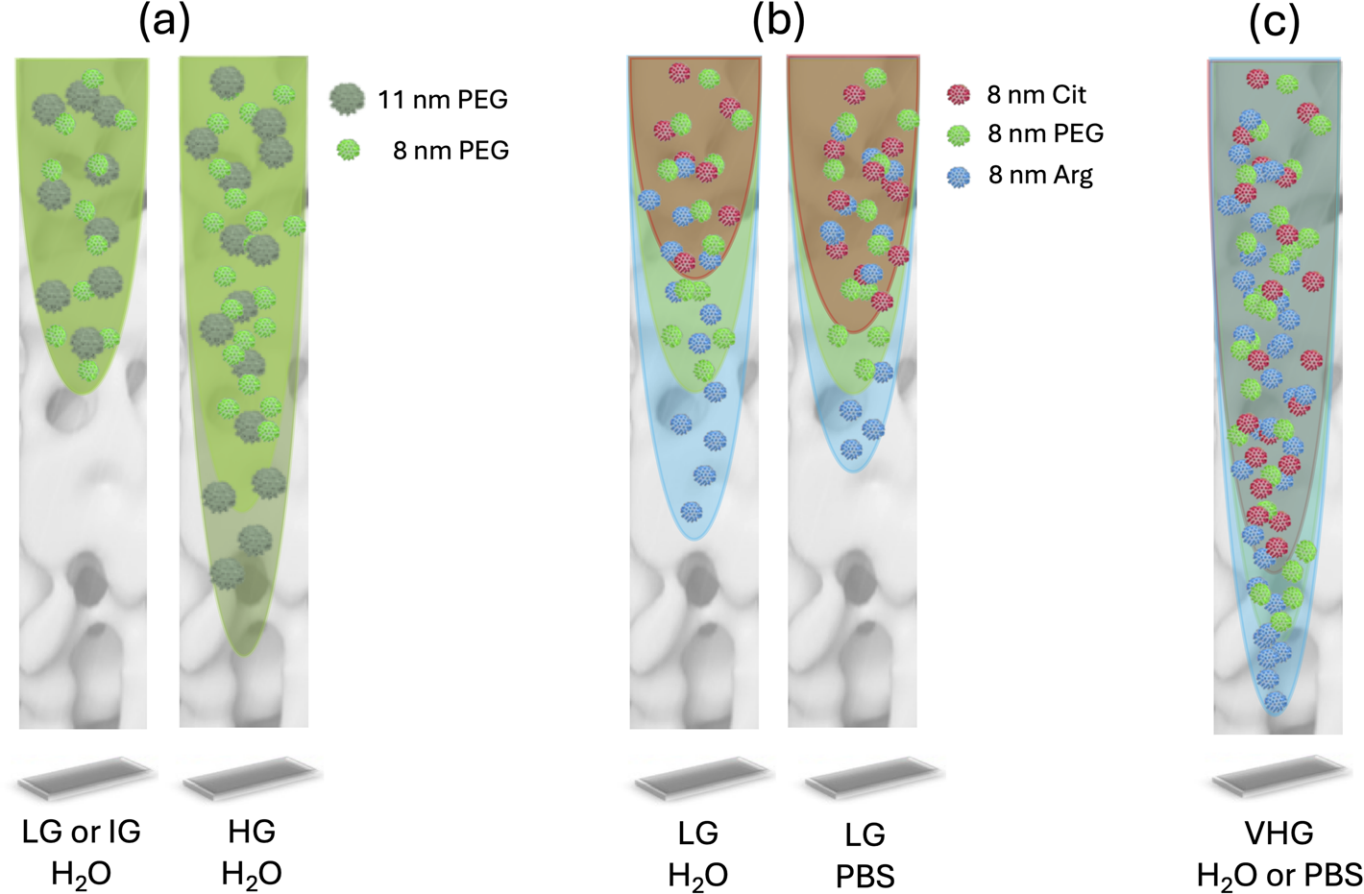
Representations of MNP transits (trends in, *v*_exp_ × *d*_hyd_) through agarose under different conditions, see text. (a) Effect of MNP size and gradient strength; (b) effect of IS for different MNP surface chemistries at LG; (c) absence of effect of IS for different MNP surface chemistries at VHG.

At low gradient the experimental velocities, *v*_exp_, for the 8 nm MNPs were very similar to those described by us previously using the same gradient for similarly-sized MNPs with the same surface chemistries.^13^ In these conditions *v*_exp_ for the larger, 11 nm MNPs was surprisingly observed to be the same, within error, as that measured for the 8 nm particles, Table 1. The *v*_exp_ values for both particle sizes were found to increase with increasing magnetic force, as expected. The effect of particle size was also found to be negligible at low and intermediate gradients (*p* > 0.05 in both cases). However, at high gradient the 11 nm MNPs achieved and maintained higher velocity throughout their transits (1.28 mm h^−1^ for 8 nm, 1.54 mm h^−1^ for 11 nm, *p* = 0.0016), which we ascribe to the larger particle moment resulting in greater deformation of the matrix at higher magnetic force. Nevertheless the effect of particle size, in this range, on *v*_exp_ is relatively weak, so size can be selected on the basis of other properties. The relative changes in the scaled velocities are represented in Scheme 1a, which emphasises the fractional increase for the larger MNPs on moving to HG. Surface binding capacity, which is important for applications in drug delivery and endogenous analyte capture^9^ increases with reducing partic+le size. Hence for the rest of this study smaller MNPs were used.

We next turn to the effect of surface chemistry on transport at different gradient strengths. In this case 8 nm MNPs (from a second batch, with similar *d*_TEM_ of 8.9 ± 0.8 nm) were grafted with PEG1000 (giving *d*_hyd_ 24.1 nm, PDI 0.16, *ζp* −9 mV at pH 7.4); arginine (28.0 nm, 0.16, +30 mV), or citrate (12.1 nm, 0.17, −27 mV), to enable assessment of the impact of surface charge. The agarose was expanded either in water or PBS (isotonic). Magnetophoresis data, acquired using VHG and LG, is shown in Fig. 1b, Tables S1 and 2.

At LG for agarose (H_2_O) the *v*_exp_ values obtained were similar for Arg-MNP and PEG-MNP, and were significantly higher for Cit-MNP, Table S1. This is expected, given the smaller *d*_hyd_ and so reduced drag of the latter. Very similar velocities were observed for the three surface chemistries using the low gradient under isotonic conditions. The scaled velocity (*v*_exp_ × *d*_hyd_) values are provided in Table 2. As described in the Introduction this scaling corrects for size-dependent differences in drag, and so the values can be more instructive. For magnetophoresis using LG through agarose (H_2_O), positively charged Arg-MNPs showed higher scaled velocity, of 9.8 × 10^−6^ mm^2^ h^−1^, than did PEG-MNPs at 8.9 × 10^−6^ mm^2^ h^−1^. We suggest this is due to attractive electrostatic interactions enhancing the transport rate through pore restrictions. Cit-MNPs, on the other hand, travelled more slowly, at 7.7 × 10^−6^ mm^2^ h^−1^, due to repulsive interactions.

At low gradient in isotonic conditions, using agarose (PBS), PEG-MNPs had unchanged scaled velocities, confirming minimal electrostatic interactions with the agarose chains. For Arg-MNPs *v*_exp_ × *d*_hyd_ was reduced by a factor of ×0.92, reaching close to the value for PEG-MNPs, indicating full suppression of the EDLs resulting in velocity being determined by the matrix tortuosity only. For Cit-MNPs the fractional change in *v*_exp_ × *d*_hyd_ for isotonic conditions of ×1.06, was not quite enough to achieve the PEG-MNP velocity. This suggests that the EDLs of Cit-MNPs are not fully collapsed under these conditions, so electrostatic pore restrictions still retard transport somewhat. Overall the effects on the EDLs of expanding agarose with PBS are similar to our previous report.^13^ The relative changes in the scaled velocities, obtained for LG, are represented in Scheme 1b, which emphasises the close similarity in the values (loss of modulation) on moving to isotonic conditions.

At VHG in agarose (H_2_O), *v*_exp_ values showed a similar pattern (to at low gradient) with higher velocity for Cit-MNPs, Table S1. Turning to the scaled velocities, the values recorded were uniformly higher than at LG, Table 2, as expected. Cit-MNPs were again found to have lower, and Arg-MNPs higher, *v*_exp_ × *d*_hyd_ than PEG-MNPs, in both DI H_2_O and under isotonic conditions, and this difference was far greater than at low gradient. Interestingly, at very high gradient the change to isotonic media had no impact on *v*_exp_ × *d*_hyd_. Fractional changes of 1.02, or less, were observed for the electrostatically-stabilised cases, as compared to 0.92 (Arg-MNPs) and 1.06 (Cit-MNPs) at low gradient. This demonstrates decreasing influence of MNP surface charge on the interactions with pore restrictions at low IS. This situation is represented in Scheme 1c, which emphasises the lack of modulation for VHG at low IS, as compared to LG, Scheme 1b. We suggest that attractive/repulsive interactions at the restrictions are simply overwhelmed. Higher magnetic force increases the number of particle collisions with the restrictions in a given time, and these are also likely to result in greater deformation of the chains reducing the barrier to transport. Consistent with this interpretation, for VHG only, after transit there is evidence of damage to the hydrogel, Fig. S3.

There are advantages to using very high gradient magnets to maximise MNP flux. However, our results show that magnetophoretic transport using lower gradients provides greater insights into particle–matrix interactions. In addition for application in labs and clinics there are significant safety issues associated with very high gradients and potential for tissue damage from rapidly transiting particles. Hence the low gradient (LG) was used for the remainder of this study.

### Magnetophoretic transport of functionalised MNPs in biomimetic matrices

#### Agarose–collagen composite hydrogels

In the first instance we evaluated magnetophoretic transport through agarose–collagen to probe the effect of including collagen. These hydrogels were prepared at 0.26 % w/v agarose and 0.14 mg mL^−1^ collagen, see Methods. Firstly, the effect on the meso-scale structuring of the matrix was evaluated by scanning electron microscopy (SEM) analysis of freeze-dried agarose (H_2_O) and agarose–collagen (H_2_O), Fig. S4. When collagen is present a more complete network is formed with fibers fused into interconnected quasi-planar structures which show reduced microporosity and with microscale structuring on a far greater length scale of 10–50 µm, as compared to <10 µm without collagen. These findings are broadly consistent with previously reported characterisation of agarose–collagen.^26^ Note that agarose (ISF_syn_) was selected for the high IS magnetophoretic velocity comparisons below, as agarose–collagen was expanded in ISF_syn_.

**Fig. 2:**
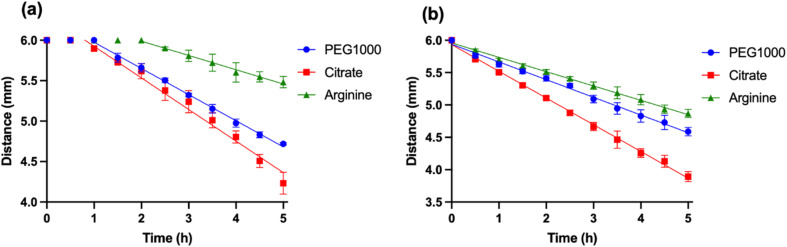
Magnetophoretic transport of 8 nm (Batch 2) PEG1000- (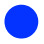), Cit- (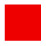) and Arg-MNP (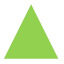) suspensions using the low field gradient (LG) through (a) agarose–collagen (ISF_syn_), at 0.5 mg mL^−1^, 0.3 % w/v, *n* = 4, *R*^2^ > 0.98 for regression model applied to the linear region (after the penetration time) only, and; (b) cultured ECM (at 1 mg mL^−1^, *n* = 4, *R*^2^ > 0.98 for the linear regression model applied to full range).

Unusually with agarose–collagen, instead of the expected spreading, upon placing the MNP suspension onto the top of the gel, the deposit remained as a spherical droplet on the surface for a period of time (up to 2 h in the case of Arg-MNPs) despite the presence of the gradient, Fig. 2a. Eventually the droplets gradually collapsed and in all cases, once the particles penetrated into the bulk normal linear transport was observed. Note that pure water droplets were rapidly absorbed/passed through unimpeded. During transit *v*_exp_ was found to be greater for Cit-MNP than for PEG-MNP, and Arg-MNP had the lowest value, Table 3. Interestingly this order of velocity correlates with the surface penetration time. We suggest that, under the gelation conditions used, hydrophobic regions of collagen may be preferentially oriented towards the air–water interface to minimise surface energy, surface dehydration effects may also contribute. Together these effects form a barrier to transport. The finding that MNPs with shorter penetration times have higher *v*_exp_ suggests that local particle dynamics are influenced by the same factors at the air interface and in the bulk. In any case, away from the surface agarose–collagen is homogeneous, as demonstrated by subsequent linear transport.

**Table 3:** Scaled *v*_exp_ values for 8 nm PEG-, Arg- and Cit-MNP suspensions through agarose (H_2_O); agarose (ISF_syn_); agarose–collagen (ISF_syn_), and; ECM under LG, see Table S2 for *v*_exp_ values

Surface chemistry	*v*_exp_ × *d*_hyd_^a^ (×10^6^ mm^2^ h^−1^)			
	Agarose (H_2_O)^b^	Agarose (ISF_syn_)	Agarose–collagen (ISF_syn_)	ECM
PEG1000	8.9	8.9	7.7(×0.86)^c^	6.4(×0.72)^c^
Arginine	9.8	8.8	4.3(×0.49)^c^	6.2(×0.70)^c^
Citrate	7.7	8.2	5.5(×0.67)^c^	5.0(×0.61)^c^

a 8 nm MNPs from Batch 2. Suspensions were at ∼1 mg mL^−1^; *n* = 4; *R*^2^ > 0.98.

b Data from Table 2.

c The (× values) are fractional changes in *v*_exp_ × *d*_hyd_, relative to agarose (ISF_syn_).

During transit the velocity through agarose–collagen is reduced for all surface chemistries, as compared to agarose (H_2_O) or agarose (ISF_syn_), Table S2. For this matrix the *v*_exp_ × *d*_hyd_ values are, again, more useful; for PEG-MNPs the reduction in scaled velocity is by a factor of ×0.86, as compared to in agarose (ISF_syn_), Table 3. This is consistent with a more tortuous path/some engagement with the collagen despite the stable hydrated layer on the MNPs. For electrostatically stabilised MNPs of either surface charge the scaled velocity is more strongly reduced. This effect is stronger for Arg-MNPs, for which *v*_exp_ × *d*_hyd_ decreases by a factor of 0.49 compared to agarose (ISF_syn_), far greater than the 0.67 fold decrease for Cit-MNPs. This behaviour is unlike the increased and decreased velocity observed in agarose for Cit-MNP and Arg-MNP, respectively, on moving from low to high IS. Quartz crystal microbalance measurements have previously shown that both positively and negatively charged Au NPs have net attractive interactions with collagen.^31^ Apparently similar attractive interactions are present in agarose–collagen for both Cit-, and particularly Arg-MNPs, that determine velocity even at this relatively high IS. The transit for Arg-MNPs in agarose–collagen, as measured by *v*_exp_ × *d*_hyd_, is the slowest observed in the entire study, even when using ECM as the matrix.

#### Extracellular matrix

In the case of transport through ECM, no delayed penetration effects were observed for any MNP surface chemistry and the transits were again found to be linear throughout, Fig. 2b, indicating similar viscoelastic effects as for agarose-based hydrogels. The scaled velocities for this matrix were observed to be lower than through agarose (ISF_syn_) for all MNPs, Table 3. In fact they were the lowest *v*_exp_ × *d*_hyd_ values measured, with the exception of the anomalous slow transport of Arg-MNPs through agarose–collagen, noted above. SEM analysis of freeze-dried ECM showed (as compared to agarose and agarose–collagen) a network of denser, in places thicker, interconnected fibres, with further reduced microporosity and with microscale structuring on a far greater length scale of >10 µm, as compared to <10 µm in agarose, Fig. S4. The more tortuous matrix apparently gives rise to reduced scaled velocity.

We suggest that PEG-MNPs, with minimal electrostatic effects and maximal surface hydration, interact weakly with the matrix and more clearly reflect the network tortuosity. By that measure ECM is less tortuous than agarose–collagen and both are less tortuous than agarose, Table 3. As compared to PEG-MNPs, electrostatic interactions in ECM slightly reduce *v*_exp_ × *d*_hyd_ for Arg-MNPs and significantly reduce it for Cit-MNPs. It is interesting that in the two simpler matrix types, while increasing IS fully suppressed electrostatic effects for Arg-MNPs (reducing *v*_exp_ × *d*_hyd_ to the expected value), for Cit-MNPs the equivalent enhancement was never complete. In ECM retention of some electrostatic interactions for Cit-MNP at high IS is again apparent from its slow transport.

### Comparison of magnetophoretic transport in the different hydrogels

The factors that determine magnetophoretic transport through agarose remain the most clear, largely due to the relative simplicity of the material. The effect of suppressing the EDLs by increasing IS, through whatever means, is to modulate (increased velocity for Cit-MNPs and reduced for Arg-MNPs) the particle flux through the negatively charged pore restrictions. This effect is effectively eliminated at higher field gradient, as the flux is significantly higher.

In the case of agarose–collagen, PEG-MNPs interact weakly with all matrices studied, experiencing low barriers to transport. However modulation of scaled velocity for oppositely charged particles is not observed. Instead Cit-, and particularly, Arg-MNPs have net attraction to the matrix even at relatively high IS, presumably due to electrostatic interactions with some residues in the collagenous component. This shows that firstly these MNPs do not travel unimpeded through a continuous agarose sub-network, and secondly velocity is reduced despite the more open micro-pore structure evident from SEM, *i.e.* attractive interactions with collagen determine the magnetophoretic velocity, not pore size.

For ECM the *v*_exp_ × *d*_hyd_ values for PEG-MNP transport suggest increased tortuosity. However it is more difficult to separate contributions from electrostatic interactions with the matrix formers and with the other biomolecules from the tortuosity. The EHS murine sarcoma-derived matrix used in this study is a basement membrane preparation whose principal components are collagen IV, laminin, and the heparan sulphate proteoglycan perlecan. The charged environment of this matrix at physiological pH is complex, transiting MNPs encounter both positive and negative charges, and a simple net-charge electrostatic argument is insufficient to fully rationalise the observed transport behaviour. In this context the particularly slow transport of Cit-MNPs, and its persistence at high ionic strength where Debye screening would be expected to suppress long-range electrostatic interactions, is consistent with specific adsorptive interactions, beyond simple screened electrostatics.

Although ECM contains a significant amount of negatively charged proteoglycans, which might be expected to increase/reduce velocity for Arg-/Cit-MNPs, the absence of any modulation shows that such interactions are not limiting or, more likely, they are one of several contributing interactions. The relatively slow scaled velocity of Cit-MNPs suggests, again, that at high IS some electrostatic interactions with the matrix remain. We suggest that the next step, prior to computational modelling of transport in ECM, should be to extract the ECM components and identify key interactions with the different MNP types.

## Conclusions

Magnetophoretic transport of magnetic nanoparticles reveals particle–matrix interactions in biomimetic matrices that determine magnetic capture. Particle size effects are shown to be weak and lower field gradients, of *c.* 45 T m^−1^, are found to be more useful for probing particle–matrix interactions. The scaled velocity, *v*_exp_ × *d*_hyd_, is shown to be a useful comparative measure of these interactions, or in their absence, of the matrix tortuosity. It is found that electrostatic interactions are not fully suppressed in complex matrices, at least in the case of negatively charged Cit-MNPs, while Arg-MNPs have preferential interactions with the agarose component in agarose–collagen matrices. Despite open porosity, transport over macroscopic distances is determined by the nanoscale interactions.

The findings of this study are directly applicable to transdermal drug delivery and biosensing applications involving superficial tissue layers. In other tissues, for instance solid tumours (a common target for magnetophoretic particle deposition), elevated interstitial fluid pressure may represent an additional barrier to inward transport not captured by the static hydrogel models used here. Nevertheless the dependence of magnetophoretic transport on MNP surface chemistry at physiological ionic strength, and the persistence of electrostatic and other interactions in complex matrices, are relevant to any application in which MNPs transit through collagen- and heparan sulphate-containing matrices under a magnetic field gradient.

## Conflicts of interest

There are no conflicts to declare.

## Supplementary Material

NA-008-D6NA00088F-s001

## Data Availability

Data for this article, including .csv files for all figures in main text and supplementary information (SI). Supplementary information is available. See DOI: https://doi.org/10.1039/d6na00088f.

## References

[cit1] Maniwongwichit N., Morarad R., Sakunpongpitiporn P., Parinyanitikul N., Paradee N., Sirivat A. (2024). Mater. Chem. Phys..

[cit2] Dobson J. (2006). Nanomed.

[cit3] Jang B., Amirshaghaghi A., Choi J., Miller J., Issadore D. A., Busch T. M., Cheng Z., Tsourkas A. (2025). ACS Nano.

[cit4] Friedman A. D., Claypool S. E., Liu R. (2013). Curr. Pharm. Des..

[cit5] Chatterjee D. K., Diagaradjane P., Krishnan S. (2011). Ther. Deliv..

[cit6] Liu J. F., Lan Z., Ferrari C., Stein J. M., Higbee-Dempsey E., Yan L., Amirshaghaghi A., Cheng Z., Issadore D., Tsourkas A. (2020). ACS Nano.

[cit7] Pan Y., Zhang D., Yang P., Poon L. L. M., Wang Q. (2020). Lancet Infect. Dis..

[cit8] Yarmola E. G., Shah Y., Arnold D. P., Dobson J., Allen K. D. (2016). Ann. Biomed. Eng..

[cit9] Lyons S., Baile Pomares P., Vidal L., McGarry K., Morrin A., Brougham D. F. (2023). Langmuir.

[cit10] Tan Z., Dini D., Rodriguez y Baena F., Forte A. E. (2018). Mater. Des..

[cit11] Murphy N. P., Lampe K. J. (2015). J. Mater. Chem. B.

[cit12] Holligan D. L., Gillies G. T., Dailey J. P. (2003). Nanotechnology.

[cit13] Lyons S., Kiernan E. P. M., Dee G., Brougham D. F., Morrin A. (2020). Nanoscale.

[cit14] Zimina T., Sitkov N., Brusina K., Fedorov V., Mikhailova N., Testov D., Gareev K., Samochernykh K., Combs S., Shevtsov M. (2024). Nanomaterials.

[cit15] Kuhn S. J., Hallahan D. E., Giorgio T. D. (2006). Ann. Biomed. Eng..

[cit16] Kulkarni S., Ramaswamy B., Horton E., Gangapuram S., Nacev A., Depireux D., Shimoji M., Shapiro B. (2015). J. Magn. Magn. Mater..

[cit17] Plecis A., Schoch R. B., Renaud P. (2005). Nano Lett..

[cit18] Bruno G., Di Trani N., Hood R. L., Zabre E., Filgueira C. S., Canavese G., Jain P., Smith Z., Demarchi D., Hosali S., Pimpinelli A., Ferrari M., Grattoni A. (2018). Nat. Commun..

[cit19] Arnott S., Fulmer A., Scott W. E., Dea I. C., Moorhouse R., Rees D. A. (1974). J. Mol. Biol..

[cit20] Barrangou L. M., Drake M., Daubert C. R., Foegeding E. A. (2006). Food Hydrocoll..

[cit21] Engin A. B., Nikitovic D., Neagu M., Henrich-Noack P., Docea A. O., Shtilman M. I., Golokhvast K., Tsatsakis A. M. (2017). Part. Fibre Toxicol..

[cit22] Cahn D., Stern A., Buckenmeyer M., Wolf M., Duncan G. A. (2024). ACS Nano.

[cit23] Stylianopoulos T., Poh M.-Z., Insin N., Bawendi M. G., Fukumura D., Munn L. L., Jain R. K. (2010). Biophys. J..

[cit24] Lieleg O., Baumgärtel R. M., Bausch A. R. (2009). Biophys. J..

[cit25] Ulrich T. A., Jain A., Tanner K., MacKay J. L., Kumar S. (2010). Biomaterials.

[cit26] Batorsky A., Liao J., Lund A. W., Plopper G. E., Stegemann J. P. (2005). Biotechnol. Bioeng..

[cit27] Lake S. P., Hald E. S., Barocas V. H. (2011). J. Biomed. Mater. Res., Part A.

[cit28] Pinna N., Grancharov S., Beato P., Bonville P., Antonietti M., Niederberger M. (2005). Chem. Mater..

[cit29] Ninjbadgar T., Brougham D. F. (2011). Adv. Funct. Mater..

[cit30] Fox E. , The Preparation and Characterisation of Stable Colloids of Magnetic Nanoparticles and Nanoparticle Assemblies with Controlled Size and Magnetic Resonance Properties, PhD thesis, Dublin City University, 2014

[cit31] Wang D., Ye J., Hudson S. D., Scott K. C. K., Lin-Gibson S. (2014). J. Colloid Interface Sci..

