# Supplementary material for: Magnetophoretic transport of functionalised iron-oxide nanoparticles through biomimetic hydrogels and extracellular matrix
Source: Nanoscale Adv. 2026 May 12;8(14):4008–16. doi: 10.1039/d6na00088f (PMC13248886; doi:10.1039/d6na00088f)
Supplement: NA-008-D6NA00088F-s001 [file NA-008-D6NA00088F-s001.pdf]

Supplementary Information (SI) for Nanoscale Advances.
This journal is © The Royal Society of Chemistry 2026

Supplementary Information

Magnetophoretic transport of functionalised iron-oxide nanoparticles through biomimetic hydrogels and extracellular matrix

Stephen Lyons,[a] Katie McGarry,[b] Aline F. Miller,[c] Rinki Singh,[a] Aoife Morrin[a*] and Dermot F. Brougham[b*]

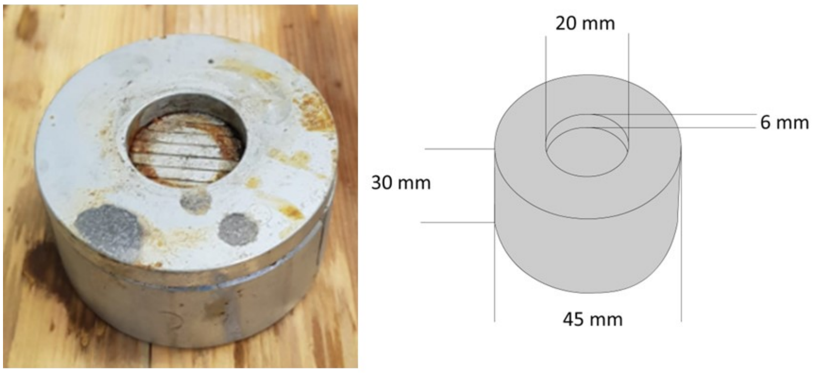

Figure S1. Left, photograph of the GIAMAG magnet. Right, the dimensions of the magnet, note that it provides magnetic force ($B.\nabla B$) of *c.*260 $T^2.m^{-1}$ at the pole face.

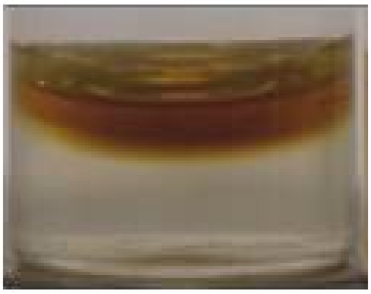

Figure S2. Image of progress of the NMR front through 0.3 %w/v agarose.

Table S1. Magnetophoretic velocities (v_{exp}) for low and very high magnetic field gradients for 8 nm PEG1000-MNP, Arg-MNP, and Cit-MNP suspensions[a] through Agarose(H_2O) and Agarose(PBS) as the isotonic condition.

	Low gradient		***Very high gradient***	
Surface chemistry	Agarose(H_2O) (Hypotonic)	Agarose(PBS) (Isotonic)	Agarose(H_2O)	Agarose(PBS) (Isotonic)
	v_{exp} (mm h^{-1})[b]	v_{exp} (mm h^{-1})	v_{exp} (mm h^{-1})	v_{exp} (mm h^{-1})
PEG1000	0.37 (±0.02)	0.37 (±0.02)	4.69 (±0.37)	4.72
Arginine	0.35 (±0.01)	0.31 (±0.02)	4.73 (±0.15)	4.75
Citrate	0.63 (±0.02)	0.67 (±0.04)	5.35 (±0.07)	5.43

[a] PEG1000-MNPs from Batch 2, d_{TEM} 8.9±0.8 nm (d_{hyd} 24.1 nm, PDI 0.16); Arg-MNPs (28.0 nm, 0.16); Cit-MNPs (12.1 nm, 0.17).

[b] Values taken from [12]; n=4; R^2>0.98 for all data sets.

Table S2. v_{exp} values for PEG-MNP, Arg-MNP and Cit-MNP suspensions through agarose(H_2O); agarose(ISF_{syn}); agarose-collagen(ISF_{syn}), and; ECM under a low magnetic field gradient.

Surface chemistry	***v_{exp}***[a] *(mm h^{-1})*			
	Agarose(H_2O)[b]	Agarose(ISF_{syn})	Agarose-collagen(ISF_{syn})	ECM
PEG1000	0.37 (±0.02)	0.37 (±0.02)	0.32 (±0.02)	0.27 (±0.03)
Arginine	0.35 (±0.01)	0.32 (±0.01)	0.16 (±0.02)	0.22 (±0.02)
Citrate	0.63 (±0.02)	0.68 (±0.03)	0.46 (±0.03)	0.41 (±0.02)

[a] 8 nm MNPs are from Batch 2. Suspensions were at ~1 mg mL^{-1}; agarose 0.3 %w/v (high EEO), n=4 ; R^2>0.98

[b] Data from Table S1.

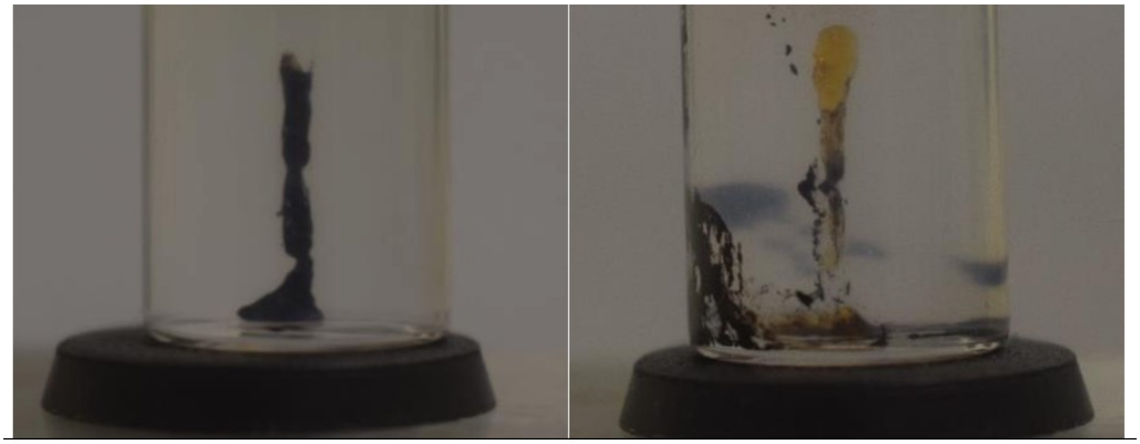

Figure S3. Left, PEG1000-MNPs in agarose/collagen ISF during magnetophoretic transport (after the front has reached the bottom of the vial, hence the spreading). Right, the same gel after all the deposit has passed through.

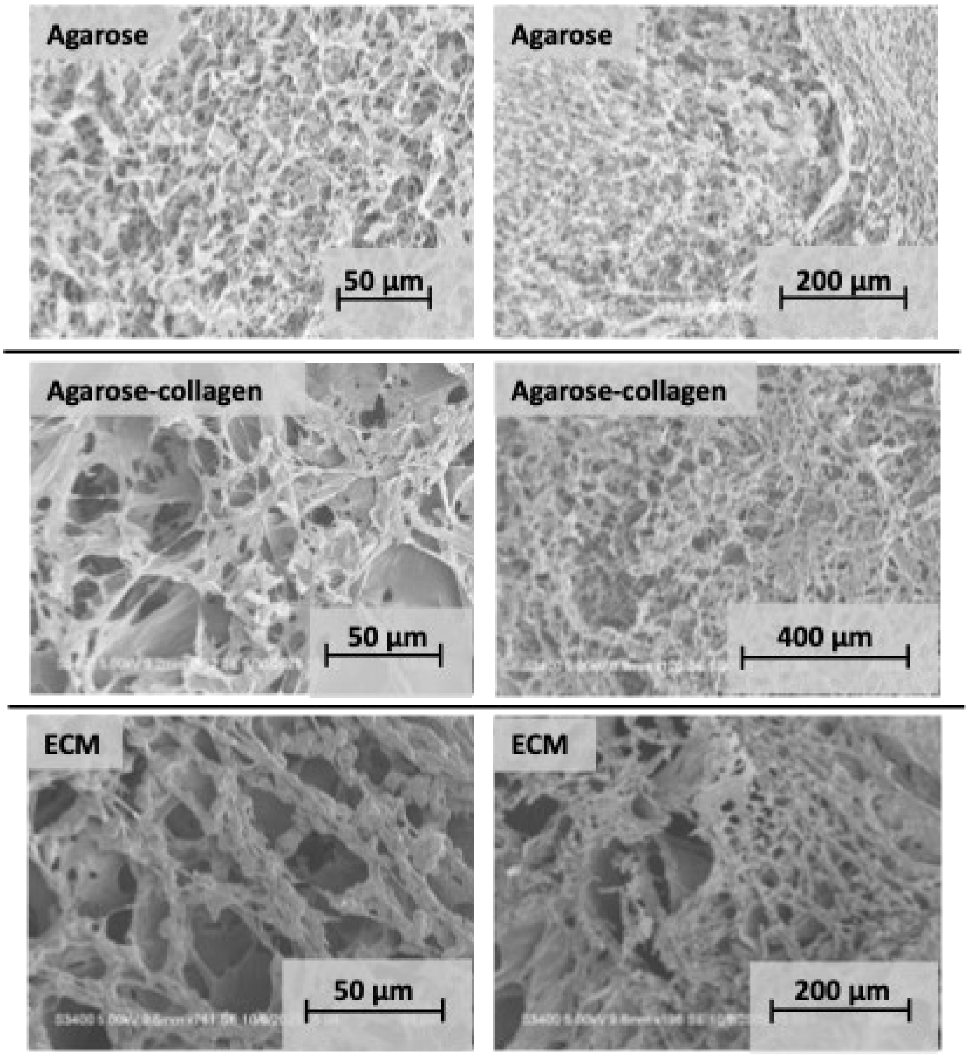

Figure S4. Selected images of agarose(H_2O) and agarose-collagen(H_2O) surface morphologies examined using SEM (Hitachi S-3400N) after freeze-drying samples at -58 °C for 48 h in a lyophiliser. Prior to lyophilisation, the gels were immersed in liquid nitrogen. Samples were gold-sputtered for analysis.
